# Rocket science: A review of phytochemical & health-related research in *Eruca* & *Diplotaxis* species

**DOI:** 10.1016/j.fochx.2018.100002

**Published:** 2018-12-22

**Authors:** Luke Bell, Carol Wagstaff

**Affiliations:** a School of Agriculture, Policy & Development, University of Reading, Whiteknights, Reading, Berkshire RG6 6AP, UK; b Department of Food & Nutritional Sciences, University of Reading, Whiteknights, Reading, Berkshire RG6 6AP, UK

**Keywords:** Arugula, Rucola, Brassicaceae, Glucosinolates, Isothiocyanates, Flavonols

## Abstract

Rocket species (*Eruca* spp. and *Diplotaxis* spp.) are becoming increasingly important leafy salad crops across the world. Numerous scientific research papers have been published in recent years surrounding the potential health benefits associated with phytochemicals contained in leaves, such as flavonoids and isothiocyanates. Other research of note has also been conducted into the unique taste and flavour properties of leaves, which can be hot, peppery, bitter, and sweet depending upon the genotype and phytochemical composition. While research into these aspects is increasing, some studies lack cohesion and in-depth knowledge of commercial breeding and cultivation practices that makes interpretation and application of results difficult. This review draws together all significant research findings in these crops over the last five years, and highlights areas that require further exploration and/or resolution. We also advise on experimental considerations for these species to allow for more meaningful utilisation of findings in the commercial sector.

## Highlights

• Recent phytochemical research in rocket species is critically reviewed.
• Glucosinolates and hydrolysis products change over growth and shelf life.
• Experiments should better consider and account for commercial practices.
• Research should be focused on providing benefits to the end consumer.

## 1 Introduction

Rocket species are commercially important salad crops that are cultivated across the world. Leaves are commonly consumed raw in salads, and are known for distinctive pungent and peppery flavours (Bell et al., 2017, Bell et al., 2017, Bell et al., 2017). *Eruca sativa* and *Diplotaxis tenuifolia* make up the bulk of worldwide cultivation (Possenti et al., 2016) and are often marketed commercially as the same product.

*E. sativa* is a morphologically diverse species, and some cultivars share similar characteristics to *D. tenuifolia* (Bell & Wagstaff, 2014). The species contain glucosinolates (GSLs): secondary metabolites that are broken down by myrosinase enzymes into isothiocyanates (ITCs) and various other compounds. These are produced and utilised by plants of the Brassicaceae family as a means of defense against pests and herbivores, and in some cases, as attractants (Müller et al., 2015). In humans, the compounds are noted for their potential anticarcinogenic activity and other health benefits.

The last ten years has seen a marked increase in scientific studies relating to the species’ phytochemistry and health related traits. Despite significant progress in understanding the diversity and behavior of rocket plants and phytochemical compounds, there are some areas of study that are lacking cohesion and robustness, and these will be evaluated with the aim of stimulating definitive experimental resolution. Recent research outputs and results will be discussed in the context of the species natural history, commercial production, and future crop improvement efforts for improved sensory quality and health traits. We will specifically address what needs to be done in terms of breeding and genomics, as well as with phytochemical research to attain these goals.

## 2 Background & description of rocket species

### 2.1 Eruca species

#### 2.1.1 Taxonomic classification

*E. sativa* is sometimes referred to as “cultivated” rocket, “annual” rocket, “true” rocket, arugula, roquette, “white pepper”, or taramira (Garg & Sharma, 2014). This species is also sometimes synonymously referred to as *E. vesicaria* subsp. *sativa* (Pasini, Verardo, Cerretani, Caboni, & D’Antuono, 2011), but the exact taxonomic classification has yet to be properly agreed, as rigorous phylogenetic studies are absent from the literature.

The *Eruca* genus is sometimes quoted as having a greater genetic diversity due to its monospecific nature (Hall et al., 2012a). This is debatable, as there are currently five species recognised by the Med-Checklist (an online inventory of vascular plants of circum-Mediterranean countries; Greuter, Burdet, & Long, 2008). These include the two aforementioned names, as well as *E. loncholoma*, *E. pinnatifida* and *E. setulosa*. Until a comprehensive genomic and phylogenetic survey is conducted, the ambiguity surrounding the diversity of the species will remain.

*E. sativa*/*vesicaria* is a diploid organism containing 11 pairs of chromosomes (Table 1, 2*n* = 22; Nothnagel, Budahn, Schrader, & Klocke, 2012). The species is a preferential out-breeder, with varying degrees of self-incompatibility between cultivars. This mechanism can be overcome by performing bud-pollinations by hand, and reducing ambient temperatures during flowering (Lou et al., 2008). Crosses with *Diplotaxis* species have been attempted with no known viable results, but somatic hybrids have been produced with *Brassica oleracea*, with the purpose of introducing cytoplasmic male sterility into the species (Carree et al., 2014).

**Table 1:** Rocket species names, observed chromosome ploidy counts, and native areas according to Med-Checklist (Greuter, Burdet, & Long, 2008) and Eschmann-Grupe, Hurka, and Neuffer (2003).

Species name	Chromosome ploidy	Geographical origin
*Eruca* spp.		
*Eruca sativa*	2n = 22	Algeria, Turkey, Spain, Bulgaria, France, Greece, Cyprus, Israel, Jordan, Italy, Libya, Lebanon, Syria, Portugal, Morocco, Malta, Ukraine, Iran, India, Pakistan
*Eruca vesicaria*		Algeria, Spain, Morocco
*Eruca loncholoma*		Algeria, Tunisia
*Eruca pinnatifida*		Algeria, Spain, Morocco, Tunisia
*Eruca setulosa*		Algeria, Morocco

*Diplotaxis* spp.		
*Diplotaxis erucoides*	2n = 14	Belgium, France, Canary Islands, Romania, Spain, Italy, Algeria, Egypt, Israel, Jordan, Serbia, Lebanon, Syria, Morocco, Malta, Tunisia
*Diplotaxis harra*	2n = 26	Egypt, Algeria, Spain, Israel, Jordan, Libya, Lebanon, Syria, Morocco, Italy, Tunisia
*Diplotaxis harra* ssp. *crassifolia*		Italy, Spain, Algeria, Morocco, Tunisia
*Diplotaxis siettiana*	2n = 16	Spain
*Diplotaxis ibicensis*		
*Diplotaxis brevisiliqua*		Morocco
*Diplotaxis catholica*	2n = 18	Spain, Portugal, Morocco
*Diplotaxis viminea*	2n = 20	Germany, Greece, Algeria, Turkey, Spain, Bulgaria, Cyprus, Egypt, France, Spain, Israel, Jordan, Italy, Serbia, Lebanon, Syria, Portugal, Morocco, Malta, Ukraine
*Diplotaxis siifoila*		Morocco, Algeria, Spain, Portugal
*Diplotaxis tenuifolia*	2n = 22	Switzerland, Germany, Netherlands, Austria, Italy, Hungary, Italy, France, Romania, Spain, Turkey, Albania, Bulgaria, Serbia, Lebanon, Syria, Portugal, Morocco, Malta, Ukraine
*Diplotaxis cretacea*		Russia
*Diplotaxis simplex*		Algeria, Egypt, Libya, Tunisia
*Diplotaxis muralis*	4n = 42	Germany, Greece, Spain, Austria, Italy, Belgium, Denmark, Algeria, Albania, Bulgaria, France, Serbia, Libya, Morocco, Malta, Ukraine, Tunisia, Turkey

The mitochondrial genome of *E. sativa* has been sequenced (Wang et al., 2014), however this did little to resolve the number of species present within the genus. It was only determined that the genus is more closely related to *B. oleracea* than *Raphanus sativus* (radish) and *Arabidopsis thaliana*. As discussed by Bell and Wagstaff (2014), the assertion that *E. sativa* is more highly domesticated than “wild” rocket (*D. tenuifolia*) has no supporting evidence.

One study by Egea-Gilabert, Fernandez, Migliaro, Martinez-Sanchez, and Vicente, (2009) looked at the genetic diversity between *E. vesicaria* and *D. tenuifolia* for agronomic traits and found a large amount of diversity within each species (using 6 ISSR markers; inter simple sequence repeats). A slightly more comprehensive analysis by Taranto et al. (2016) using ISSR markers found a higher number of polymorphisms in *Eruca* (77) than *Diplotaxis* (40), indicating perhaps that genetic variation is greater in *Eruca* species. These analyses are very limited from a genomics standpoint however, as the number of ISSR primers used is extremely small compared to the total number of potential genomic single nucleotide polymorphisms (SNPs), and not indicative of whole genome variability. As these markers cannot be linked to any specific traits of interest, their usefulness to breeders is very limited.

#### 2.1.2 Natural history & cultivation

Regardless of the speciation status of the *Eruca* genus, *E. sativa* is noted for having a fast growing nature, and for the hotness (pungency) and pepperiness of its leaves (Pasini et al., 2011). This is reflected in the latin name, which originates from “uro” or “urere”; which translates to burn in English. Historically *E. sativa* has been grown in the countries and regions surrounding the Mediterranean Sea, and use can be traced back to Roman times. A common use for the plant was as an aphrodesiac, and is regularly referenced in ancient texts for such properties (Hall et al., 2012b). There is no scientific evidence to support this property of leaves however.

The species’ natural ecological distribution covers southern Europe, north Africa, Iran, India and Pakistan, and is traditionally grown as a winter crop in dry areas. It has evolved a fast-growing and efficient root system, and is capable of withstanding severe drought conditions. This property makes it an important traditional food source in arid areas (Garg & Sharma, 2014). Due to its weedy and hardy nature, the species has become naturalised on every permanently inhabited continent. In Western countries, it is now most commonly used as a salad or garnish (Jin et al., 2009). Leaves are sold in both processed and fresh markets (Hall, Jobling, & Rogers, 2013) and the crop is therefore gaining significant economic importance (Hall et al., 2012a). In India and Pakistan, *Eruca* species are also used widely as oliseed, forage and fodder crops. Roots, flowers and seeds are all consumed and processed in a similar fashion to how mustard species are in Western countries (Garg & Sharma, 2014).

### 2.2 Diplotaxis species

#### 2.2.1 Taxonomic classification

Unlike *Eruca* species, there is greater consensus about the diversity of the *Diplotaxis* genus. It is agreed that it is polyphyletic (Table 1), but that morphology is an inadequate means of determining evolutionary relatedness (Arias & Pires, 2012). *Diplotaxis* species are diploid, with one exception; *Diplotaxis muralis* is thought to be an amphiploid of *D. tenuifolia* and *D. viminea*, and it is the only known tetraploid species within the genus (Eschmann-Grupe et al., 2003). *D. tenuifolia* contains 11 pairs of chromosomes (2*n* = 22) like *E. sativa*. *D. erucoides* has the smallest chromosome ploidy of the genus, and it is speculated that it may represent an ancestral species (Martín & Sánchez-Yélamo, 2000).

Within-species diversity is not well established, with some authors arguing for large amounts of genetic variation (Hall et al., 2012a), however this is not substantiated by much experimental evidence. To date it has only been inferred from differences between morphological and phytochemical traits, and limited Random Amplification Polymorphic DNA (RAPD) marker analyses, which are notorious for not being reproducible (Qiao et al., 2007).

#### 2.2.2 Natural history & cultivation

*Diplotaxis* species are synonymously referred to as rocket, as with *Eruca* species. *D. tenuifolia* can be more specifically referred to as “perennial wall rocket” and is the predominant species cultivated in this genus (Hall et al., 2012a). It is similarly known for its peppery and pungent flavours (Pasini et al., 2011), and is arguably the most important species economically due to the prevalence of its commercial growth in Europe and Australia (Hall et al., 2012a).

As with *Eruca*, *Diplotaxis* species are also native to the countries surrounding the Mediterranean Sea, as well as India, Pakistan, Cape Verde and Nepal (Hall et al., 2012b). *D. harra* and *D. simplex* are common in Tunisia and North Africa, where traditionally, plants have been used for medicinal purposes because of reported antimicrobial properties, as well as for general food consumption (Falleh et al., 2013).

In terms of growth habit, *D. tenuifolia* is much slower to establish and grow than *E. sativa*. *D. erucoides* however comprises attributes of both these species, having the early vigour of *E. sativa* and the distinctive serrated-lobed leaf shape of *D. tenuifolia*, making it an attractive choice for growers.

## 3 Experimental studies of rocket species relating to phytochemical, sensory, & health promoting effects

### 3.1 General

Within the recent scientific literature several studies have been conducted in relation to phytochemical diversity of rocket species (Jin et al., 2009, Martinez-Sanchez et al., 2007, Pasini et al., 2012). Bennett and colleagues published papers in the previous decade (Bennett, Carvalho, Mellon, Eagles, & Rosa, 2007), which lay an important foundation in identifying the core GSL components of rocket. The same can be said of Martinez-Sanchez and colleagues (Martinez-Sanchez et al., 2008) in identifying polyglycosylated flavonols. Very few studies have been conducted on sensory aspects however (Bell et al., 2017, Bell et al., 2017, Bell et al., 2017, D’Antuono et al., 2009, Lokke et al., 2012, Pasini et al., 2011), and only one on consumer preferences and perceptions (Bell, Methven, & Wagstaff, 2017). Similarly, only a single study has explored the supply chain of rocket in a commercial environment (Bell, Yahya et al., 2017).

It is important to characterise rocket varieties from the perspective of the consumer. Research questions should be addressed from this standpoint, to understand the impacts postharvest processing and storage conditions have on sensory attributes, liking, and phytochemical changes. The diversity and/or abundance of GSLs/glucosinolate hydrolysis products (GHPs) and flavonols in any given variety may greatly impact the eating experience of the consumer, and it is apparent from recent findings that it is not sufficient to characterise varieties at a single time point due to the temporally dynamic nature of GSL biosynthesis and turnover (Bell, Yahya et al., 2017).

Many studies make certain assumptions about commercial practices, sensory attributes (particularly in relation to consumer preference), the composition of GSLs/GHPs, and other phytochemicals. These and other topics will be discussed with a view to reconciling misconceptions, and highlighting work where methods can be shared and adapted to advance the improvement of these species.

### 3.2 Experimental aims & designs

Several groups have published works relating to LC-MS/MS analysis of rocket GSLs, but the experimental designs of some experiments have been flawed. Publications have generally failed to take into account the changing GSL profile of plants over the course of their life cycle, or have not sampled at a commercially relevant growth stage (Bell et al., 2015). This has made consensus on exact representative GSL composition at a commercially and nutritively relevant time point (i.e. at the point of consumer ingestion) difficult. The number of accessions studied is generally small, and the characterisation of cultivars across environments and research groups is largely non-existent. Such experiments will be required in future with robust experimental designs and methods with independent cross-validation to determine trait stability and/or variation.

### 3.3 Phytochemical extraction, analysis & identification

#### 3.3.1 Glucosinolates

Determining the abundance of GSLs at any given point the rocket life cycle and postharvest treatment offers insight into how metabolic changes may influence the health beneficial effects upon consumption. Since GSL standards are still frustratingly difficult to obtain, and/or are extremely expensive, it is generally agreed that mass spectrometry is a fundamental requirement for identification. Better still is the use of MS/MS or NMR analyses, which allow confirmation and elucidation of molecular structures (Ares et al., 2015). UPLC-MS methodologies have been developed with software for the identification of GSLs (Sun et al., 2016) which could prove extremely useful where high throughput and automation is required. Some of the identifications made by this software in rocket species, particularly in regards to glucosativin and distinguishing it from glucoiberverin (both *m*/*z* 406), need to be re-evaluated to ensure the correct representation of GSL profiles is reported. This is also important for accurately attributing sensory properties to GSL-derived compounds, or indeed the GSLs themselves. False identification may lead to erroneous conclusions regarding the effects on taste, flavour, and consumer acceptance.

GSL ion data are now widely available within the literature and have been previously collated and reported clearly for rocket; for an excellent example, see Cataldi, Rubino, Lelario, and Bufo (2007). Despite the availability of such resources, many studies have failed to quantify major GSL components of rocket, such as glucosativin. The absence of this compound in published data cannot be explained by spurious reasoning, such as “cultivar variability”. It has been shown consistently in studies of large numbers of rocket accessions/cultivars that glucosativin is ubiquitous (Bell et al., 2015, Ku et al., 2016, Pasini et al., 2012, Taranto et al., 2016), and its presence (in dimer or monomer form) should therefore be used as a validation for comprehensive GSL analysis. Recent studies on the sensory properties of glucosativin and its hydrolysis product (1,3-thiazepane-2-thione, sativin; Fechner et al., 2018) have shown that it is responsible for the distinctive aroma and flavour of rocket (Raffo et al., 2018) and to fail in reporting concentrations is to completely disregard this sensory importance. This is a key compound that also determines aspects of consumer liking (Bell, Methven, & Wagstaff, 2017).

**Fig. 1:**
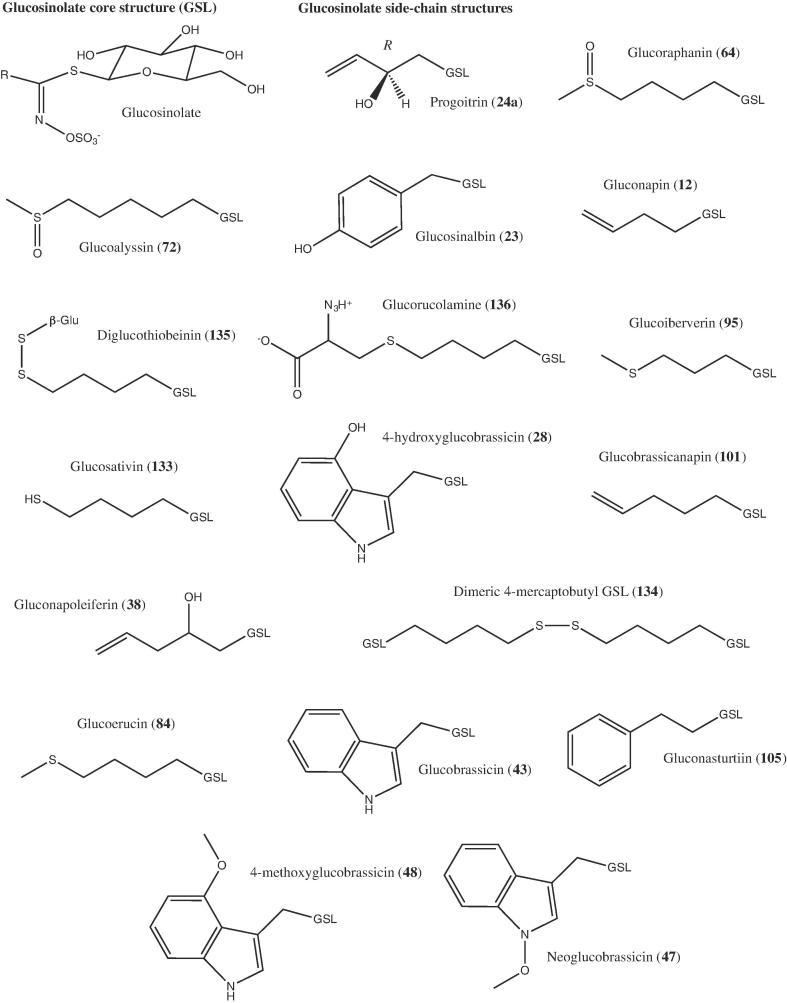
Side-chain chemical structures of glucosinolate compounds found in rocket species. Numbers in bold refer to those found in Agerbirk and Olsen (2012).

Table 2 summarises studies conducted on rocket GSLs and the concentrations/profiles that have been reported. It is widely accepted that the main constituents are glucosativin, DMB (dimeric 4-mercaptobutyl), glucoraphanin, and glucoerucin (Avato & Argentieri, 2015). As will be discussed in Section 3.4 glucosativin should also be divided into its monomer and dimer (DMB) forms where both are detected. The side-chain structures of the major and minor GSLs found in rocket species are presented in Fig. 1. Other common, but typically minor GSL constituents, include: glucoalyssin, progoitrin, 4-hydroxyglucobrassicin, diglucothiobeinin, glucorucolamine, and 4-methoxyglucobrassicin (Table 2; Bell et al., 2015). While these compounds do not occur in large concentrations, they may be important contributors to sensory attributes and consumer liking, particularly during shelf life storage.

**Table 2:** Summary of glucosinolate content of *Eruca sativa* as reported in recent studies. Concentrations are expressed as mg.g^−1^ dw of sinigrin and the values presented represent the average concentrations of all tested cultivars.

Reference	No. of cultivars tested	Cultivation Environment	Time Point of Sampling	Days After Sowing	Progoitrin	Glucoraphanin	Glucoalyssin	Glucosinalbin	Gluconapin	Diglucothiobeinin	Glucoiberverin	Glucosativin	4-Hydroxyglucobrassicin	Glucobrassicanapin	Gluconapoleiferin	Dimeric glucosativin	Glucoerucin	Glucobrassicin	Gluconasturtiin	4-Methoxyglucobrassicin	Neoglucobrassicin	Total
Bennett et al. (2007)	**34**	**CE**	**PH**	**4**–**8**	nd	**1.2**	nd	nd	nd	nd	nd	**3.6**	nd	nd	nd	**nr**	**2.9**	tr	nd	nd	nd	**7.8**
Bell, Yahya, et al. (2017)	**5**	**F**	**PH**	**12**	nd	**1.1**	nd	nd	nd	**0.2**	tr	**0.4**	nd	nd	nd	**0.1**	**0.1**	nd	nd	nd	nd	**1.8**
Bell et al. (2015)	**28**	**CE**	**H**	**30**	nd	**0.2**	tr	nd	nd	tr	tr	**3.9**	tr	nd	nd	**2.2**	**0.2**	nd	nd	nd	nd	**6.7**
Selma et al. (2010)	**1**	**CE**	**H**	**35**	nd	**4.6**	nd	nd	nd	nd	nd	**10.8**	nd	nd	nd	**nr**	**2.9**	nd	nd	nd	nd	**18.3**
Jin et al. (2009)	**1**	**CE**	**H**	**56**	nd	**0.3**	nd	nd	nd	**1.4**	**0.7**	**4.2**	nd	nd	nd	**nr**	tr	nd	nd	nd	nd	**6.6**
Pasini et al. (2012)	**32**	**F**	**H**	**?**	**0.1**	**0.5**	tr	tr	nd	**0.1**	nd	**nr**	tr	nd	nd	**0.5**	**0.3**	tr	nd	nd	nd	**1.6**
Ku et al. (2016)	**39**	**G**	**H**	**30**	tr	**2.6**	nd	nd	tr	**0.7**	nd	nd	tr	nd	nd	**7.5**	**1.8**	tr	nd	tr	nd	**13.7**
Chun et al. (2015)	**1**	**G**	**H**	**63**	nd	**0.8**	tr	nd	nd	**0.2**	nd	**nr**	nd	**0.1**	nd	**2.5**	**1.9**	tr	nd	**0.3**	nd	**6.7**
Chun, Arasu, Lim, and Kim (2013)	**15**	**G**	**H**	**69**	nd	**0.8**	nd	nd	nd	**0.1**	nd	nd	nd	tr	nd	**5.0**	**1.0**	tr	**0.1**	**0.3**	nd	**7.9**
Taranto et al. 2016	**21**	**G**	**H**	**42**–**70**	**0.8**	**2.8**	**0.6**	nd	nd	**6.8**	nd	**3.3**	nd	nd	nd	**9.8**	**5.4**	nd	nd	nd	nd	**29.5**
Kim and Ishii (2006)	**1**	**Hy**	**H**	**49**	nd	**0.5**	nd	nd	nd	**0.3**	nd	**nr**	nd	nd	nd	**2.3**	**1.3**	nd	nd	nd	nd	**4.4**
Bell, Yahya, et al. (2017)	**5**	**F**	**H**	**22**	nd	**1.0**	nd	nd	nd	**0.1**	tr	**0.7**	nd	nd	nd	**0.1**	**0.6**	nd	nd	nd	nd	**2.4**
	**5**	**F**	**PT**	**25**	nd	**1.4**	nd	nd	nd	**0.1**	tr	**1.1**	nd	nd	nd	tr	**0.8**	nd	nd	nd	nd	**3.4**
	**5**	**F**	**PR**	**26**	nd	**1.0**	nd	nd	nd	**0.1**	nd	**1.6**	nd	nd	nd	**0.2**	**0.8**	nd	nd	nd	nd	**3.6**
	**5**	**F**	**PW**	**26**	nd	**1.7**	nd	nd	nd	**0.2**	nd	**3.4**	nd	nd	nd	**0.3**	**1.3**	nd	nd	nd	nd	**6.8**
	**5**	**F**	**SL0**	**27**	nd	**1.7**	nd	nd	nd	**0.2**	tr	**3.3**	nd	nd	nd	**0.6**	**0.4**	nd	nd	nd	nd	**6.0**
	**5**	**F**	**SL2**	**29**	nd	**1.8**	nd	nd	nd	tr	nd	**3.1**	**0.1**	nd	nd	**0.7**	**1.1**	nd	nd	nd	nd	**6.8**
	**5**	**F**	**SL5**	**32**	nd	**1.8**	nd	nd	nd	**0.1**	tr	**2.3**	nd	nd	nd	**0.6**	**1.0**	nd	nd	nd	nd	**5.9**
	**5**	**F**	**SL7**	**34**	nd	**1.4**	nd	nd	nd	tr	tr	**4.1**	nd	nd	nd	**1.0**	**0.6**	nd	nd	nd	nd	**7.1**
	**5**	**F**	**SL9**	**36**	nd	**1.2**	nd	nd	nd	**0.1**	**0.1**	**1.2**	nd	nd	nd	**0.7**	**0.7**	nd	nd	nd	nd	**3.9**
Abbreviations: CE, Controlled Environment; F, Field; G, Glasshouse; ?, unknown; nd, not detected; nr, not reported; tr, trace amount; PH, Pre-harvest; H, harvest; PT, Post harvest transport; PR, Pre-washing; PW, Post-washing; SL*n*, Shelf life day.																						

Reference	No. of cultivars tested	Cultivation Environment	Time Point of Sampling	Days After Sowing	Progoitrin	Glucoraphanin	Glucoalyssin	Diglucothiobeinin	Glucoiberverin	Glucosativin	4-hydroxyglucobrassicin	Dimeric glucosativin	Glucoerucin	Glucobrassicin	Gluconasturtiin	4-methoxyglucobrassicin	Total
Bennett et al. (2007)	**3**	**CE**	**PH**	**4**–**7**	nd	**0.4**	nd	nd	nd	**9.4**	nd	**nr**	**0.3**	tr	nd	nd	**10.1**
Bell et al. (2015)	**7**	**CE**	**H**	**30**	nd	**0.2**	nd	nd	tr	**2.4**	tr	**4.7**	**0.2**	nd	nd	nd	**7.7**
Jin et al. (2009)	**1**	**CE**	**H**	**56**	nd	**0.4**	nd	**1.1**	**0.9**	**3.6**	nd	**nr**	**0.8**	nd	nd	nd	**6.8**
Pasini et al. (2012)	**5**	**F**	**H**	**?**	**0.2**	**0.4**	tr	**0.1**	nd	**nr**	**0.1**	**0.5**	**0.4**	tr	nd	nd	**1.7**
Chun et al. (2013)	**1**	**G**	**H**	**69**	nd	**1.1**	nd	**0.1**	nd	**nr**	nd	**2.8**	**1.7**	tr	**0.1**	**0.3**	**6.2**
Taranto et al. (2016)	**16**	**G**	**H**	**42**–**70**	**0.4**	**4.6**	**0.8**	**3.5**	nd	**2.0**	nd	**5.5**	**2.2**	nd	nd	nd	**19.0**
Bell, Yahya, et al. (2017)	**1**	**F**	**PT**	**25**	nd	**1.9**	nd	**0.5**	nd	**5.4**	nd	tr	**0.1**	nd	nd	nd	**8.0**
	**1**	**F**	**PR**	**26**	nd	**2.1**	nd	**0.2**	nd	**2.5**	nd	**0.6**	**1.4**	nd	nd	nd	**7.0**
	**1**	**F**	**PW**	**26**	nd	**1.8**	nd	**0.6**	nd	**2.9**	nd	**0.5**	nd	nd	nd	nd	**5.8**
	**1**	**F**	**SL0**	**27**	nd	**1.6**	nd	**0.5**	tr	**1.5**	nd	**0.5**	nd	nd	nd	nd	**4.1**
	**1**	**F**	**SL2**	**29**	nd	**0.7**	nd	**0.2**	nd	**7.1**	nd	**0.9**	nd	nd	nd	nd	**9.0**
	**1**	**F**	**SL5**	**32**	nd	**1.4**	nd	**0.2**	tr	**7.4**	nd	**0.1**	**0.5**	nd	nd	nd	**9.6**
	**1**	**F**	**SL7**	**34**	nd	**1.3**	nd	nd	nd	**7.6**	nd	**2.2**	**0.5**	nd	nd	nd	**11.5**
	**1**	**F**	**SL9**	**36**	nd	**1.5**	nd	**0.1**	nd	**5.6**	nd	**0.4**	**0.8**	nd	nd	nd	**8.5**

Abbreviations: CE, Controlled Environment; F, Field; Hy, Hydroponic; ?, unknown; nd, not detected; nr, not reported; tr, trace amount; PH, Pre-harvest; H, harvest; PT, Post harvest transport; PR, Pre-washing; PW, Post-washing; SLn, Shelf life day.

There is a high degree of genetic and environmental variability in terms of biosynthesis and accumulation of individual GSLs (Table 2), and has been observed in other species such as mustard (Kim et al., 2016), Chinese cabbage (Lee et al., 2014), green cabbage, and red cabbage (Park et al., 2014). This is an important consideration for any prospective rocket-breeding program seeking to enhance GSL/GHP profiles and sensory characteristics.

#### 3.3.2 Flavonols

The major flavonols of rocket are quercetin, kaempferol and isorhamnetin glycosides. *Eruca* species typically contain greater amounts of kaempferol, and *Diplotaxis* quercetin (Avato & Argentieri, 2015). This has been suggested as a possible means of distinguishing the two genera phytochemically, as GSL profiles are largely indistinguishable (Bell et al., 2015). Some studies have only characterised the aglycones present in rocket leaves, as this is a relatively simplistic method that can be done using HPLC and available aglycone standards. Characterising the glycosides is much more challenging, and requires an MS/MS fragmentation method to properly identify each respective compound. It was achieved to a high standard by Martinez-Sanchez et al. (2007) and has yet to be surpassed in terms of quality and detail. Properly characterising the glycoside abundance and diversity may provide a more accurate picture of the phytochemical profile; thus assisting in determining potential health promoting effects. Some glycosides are known to be more bioavailable than others after consumption, for example (Manach et al., 2005), and so breeding and selection for accumulation of specific side-chains could enhance these properties.

Bell et al. (2015) also identified many of the glycosides found by Martinez-Sanchez et al. (2007) and found that some quercetin and kaempferol glycosides were common to both rocket genera, whereas previous reports suggested that they were exclusive to one or the other. Astragalin, isoquercetrin and isorhamnetin-3-glucoside were observed in some *D. tenuifolia* cultivars; these compounds were previously only detected in *E. sativa*. Three quercetin glycosides (quercetin-3,3,4′-triglucoside, quercetin-3,4′-diglucoside-3′-(6-caffeoyl-glucoside), and quercetin-3,4′-diglucoside-3′-(6-sinapoyl-glucoside)) were detected in *E. sativa* (previously only observed in *D. tenuifolia*) and were confirmed by ion trap MS/MS fragmentation patterns. The broad trend observed was as previously reported – kaempferol was greater in abundance in *Eruca*, and quercetin in *Diplotaxis*; and Principal Component Analysis (PCA) of the data confirmed this separation.

The only other study to characterise flavonol glycosides in detail was Taranto et al. (2016) and they did not observe this cross genera presence of respective glycosides. The cultivation method was field-based, whereas Bell et al. (2015) conducted their experiment in controlled environment, which may account for some of the differences observed in total concentrations. Taranto et al. (2016) stated that differences observed were because of genetic variability within the accessions tested by Bell et al. (2015) and that their collection was “uniform”, as cultivars were “maintained in purity conditions within germplasm collections”. This assessment unfortunately misunderstands what purity conditions are when relating to gene bank accessions: purity conditions preserve the genetic diversity of collected accessions via controlled open pollinations (i.e. between numerous plants of the same accession). This therefore does not mean that these plants are genetically uniform, as would be achieved through conventional plant breeding selections and single-seed-descent – quite the opposite in fact. Such practices are specifically designed to preserve genetic diversity for breeders to utilise, not reduce it (FAO, 2014). In each of the respective studies, cultivars were obtained from the same gene banks (IPK Gatersleben, Germany; and CGN, Wageningen, The Netherlands), and so the hypothesis presented by Taranto et al. (2016) is not likely. It is perhaps more due to the large differences in the experimental environment and cultivation methods selected for plant growth.

### 3.4 Identification & quantification of dimeric glucosinolates in rocket species

A significant area of importance that needs to be reconciled within the literature is the acknowledgement of the unique dimeric GSLs found in rocket species, and their possible influence on organoleptic properties. Since the confirmation of the existence of 4-mercaptobutyl-GSL (glucosativin), the presence of dimeric GSLs have often been dismissed as extraction artifacts due to an untested hypothesis presented by Bennett et al. (2002). An experiment by Cataldi, Rubino, Lelario, and Bufo (2007) suggested however, that DMB and diglucothiobeinin are likely to be naturally occurring within leaves. Recently Fechner et al. (2018) highlighted that the formation of sativin and bis(4-isothiocyanatobutyl)-disulfide is from both hydrolysis of the respective GSLs as well as oxidation/reduction, and this does not preclude the independent stable existence and biosynthesis of dimeric GSLs *in planta*.

Despite the data presented by Cataldi et al. (2007), relatively few research papers have since cited or acknowledged this key outcome, or indeed conducted repeat experiments to determine its veracity. Several subsequent papers have disregarded detections of DMB on the basis of previous speculations and assumptions, and potentially missed important details in the genetic variability of GSL and GHP profiles between cultivars. The natural occurrence of these compounds has been noted and addressed in a prominent and comprehensive GSL structure review by Agerbirk and Olsen (2012), yet the trend for dismissal persists.

### 3.5 Identification & quantification of glucosinolate hydrolysis products

Data relating to the GHPs produced by rocket species is sparse within the literature. Much of the identification of ITCs and nitriles is derived from those common to *B. oleracea*, such as sulforaphane (SF), erucin, and their respective nitriles. Cerny, Taube, and Battaglia (1996) first described the novel ITCs and degradation products of rocket GSLs. This was recently amended and improved in a study by Fechner et al. (2018) that demonstrated sativin is in fact 1,3-thiazepane-2-thione. The ITC of glucosativin is unstable and spontaneously rearranges into a seven-carbon ring structure. This new structural elucidation potentially has large implications for the sensory and health properties of the compound.

Arora et al., 2014, Arora et al., 2016 produced papers comparing differing extraction and GC–MS conditions on the yield and abundance of erucin and other GSL hydrolytic products from rocket seeds. These data suggest that the means by which compounds are extracted and analysed has a dramatic effect on the observed profile, and that this may also be true of compounds extracted from leaf material. Fechner et al. (2018) also tested the stability of *E. sativa* hydrolysis products according to pH and duration of extraction from fresh material. These data highlighted that the compounds vary greatly in stability, with some increasing (sativin) and reducing (erucin, sulforaphane, and bis(4-isothiocyanatobutyl)-disulfide) markedly over time. This means extraction time and GC–MS settings must be considered carefully to avoid losses of important hydrolysis products such as erucin and SF. Although SF does not contribute to the aroma profile of rocket (Raffo et al., 2018), its accurate determination is fundamental for increasing health-related properties through plant breeding.

What exactly constitutes a typical rocket ITC profile is not well determined within the literature. This is due to a variety of factors, as the final observed profile is strongly affected by the extraction conditions (Fechner et al., 2018). These may include: extraction solvent type, pH and volatility, the duration of extraction and hydrolysis temperature, leaf homogenization method, liquid or headspace extraction, and leaf sample preparation (e.g. from freeze dried or fresh material). As hydrolysis product profiles change rapidly over time under any given variation in these conditions, it is hard to compare absolute quantities and ratios between studies. It is not clear what extraction conditions are the most representative of ‘real’ rocket GHP profiles, though this may depend greatly on the hypotheses and purpose of any given study. A headspace extraction of volatile ITCs is clearly more appropriate for studies of olfactory properties than a liquid extract that may also contain non-volatile, non-aroma generating components such as SF, for example.

In seeds, it is generally agreed that erucin is the predominant ITC, which is supported by the reported GSL profile (Cataldi et al., 2007). A paper by Ku, Kim, Jeffery, Kang, and Juvik (2016) gave an overview across 39 accessions of *E. sativa*. This revealed significant differences of hydrolysis product and GSL abundance according to genotype and geographical origin. The authors further highlighted the variability inherent to the species, but also the range of traits available to plant breeders to select from. According to their data and those of Bell, Yahya et al. (2017), the main hydrolysis products of rocket are: sativin, erucin, sulforaphane, and bis(4-isothiocyanatobutyl)-disulfide, though what causes differences in relative abundance and absolute quantities is likely to depend greatly on a combination of both pre-harvest factors and extraction conditions.

### 3.6 Characterisation of sensory properties & consumer preferences

Few studies have been conducted assessing the sensory properties of rocket leaves in a quantitative manner. Limited sensory analyses have been performed previously, and were typically conducted by small numbers of assessors/consumers. Such studies (<8 assessors for sensory evaluation and <60 individuals for consumer preference) do not conform to the ISO 8586:2012 and ISO 11132:2012 standards necessary for sensory analysis and performance monitoring. Other assessments have been limited by the number of traits evaluated; Inestroza-Lizardo, Silveira, and Escalona (2016) evaluated only appearance traits of one cultivar under different atmospheric packaging conditions, for example. This paper demonstrated potential benefits for using novel atmospheric compositions for preserving appearance and reducing microbial growth; but as previously pointed out, the variability between cultivars of rocket is high, and therefore the results are not representative of the species. Such studies should attempt to include as many cultivars as is practicable in order to assess the genotypic variability of such responses.

Bell, Methven, and Signore et al. (2017) conducted the most comprehensive sensory analysis of seven *E. sativa* accessions to-date, in combination with several chemical analyses. This has elucidated some of the associations between specific traits of rocket, such as GHPs and volatile sulfur compounds with hotness and mustard flavours. Other relationships that were observed have been previously unreported, such as the effects of green-leaf volatiles and free amino acids inferring a reduction in perceived pungency, and the ratio between free sugars and GSLs/GHPs reducing the perceptions of bitterness and hotness. Larger studies on an increased number of cultivars (commercial and germplasm) would aid in verifying these data, and further understanding the complex relationships between compounds in creating the distinctive flavour of rocket. A recent paper published by Raffo et al. (2018) has confirmed some of these volatile associations, as well as corrected some of the identifications made by Bell, Spadafora, Müller, Wagstaff, and Rogers (2016). Sativin is the predominant driver of rocket aroma and flavour, and it is apparent that many other compounds have subtle effects on the aroma profile of leaves.

Lokke et al. (2012) also provided a characterization of key rocket sensory aromas along with the effects of some postharvest conditions. A key outcome of this study was that using modified atmospheric packaging (MAP) helps to preserve fresh aromas in rocket, and prevent the onset of rotten odours through anaerobic respiration. This study, and that of Bell, Methven, and Signore et al. (2017), are the only sensory evaluations of rocket conducted to ISO standards to date.

Consumer data relating to preference and perceptions within the literature is largely absent. The only study conducted to-date (Bell, Methven, & Wagstaff et al., 2017) described three ‘types’ of rocket consumer: those who preferred mild/sweeter attributes, those who preferred hot (but not extremely hot) sensations, and those who broadly disliked all (but slightly favored mild/sweet) leaves. These data were correlated with phytochemical and sensory results via PCA and it was found that taste liking was largely determined by the degree of hotness within leaves, not bitter taste as had previously been assumed. This relationship was scrutinised by determining the TAS2R38 bitter taste receptor diplotype of the consenting individuals involved in the experiment. These data confirmed that homozygous ‘supertasters’ (PAV/PAV) perceived bitterness to a greater degree than non-tasters (AVI/AVI), and that the latter individuals had a propensity (though not significant) for preferring rocket more than the former. The lack of significant differences in liking between each diplotype was attributed to the influence of other factors, such as life experiences and the density of fungiform papillae.

The study provided some evidence to suggest that bitterness of rocket leaves is not a strong determining factor of liking for consumers, and that the reasons for preference are much more complex than has been previously assumed. As with sensory analysis, much more research is needed with regards to the consumer, and on a wider variety of cultivars of both *Eruca* and *Diplotaxis* species. In future it will be important to test varieties bred for enhanced GSLs/GHPs in this manner, to determine how sensory properties are affected by phytochemical changes at the point of harvest, as well as after washing, processing and storage.

### 3.7 Understanding shelf life responses to the commercial supply chain

#### 3.7.1 General

How rocket leaves respond phytochemically to commercial processing and shelf life conditions have only recently been investigated, despite the increased commercial interest and importance of the crops over the last 25 years. It is known that some cooking and preparation methods can adversely affect GSL and GHP composition (Deng et al., 2015), and in some instances show increases (Possenti et al., 2016); but rocket is usually consumed raw, and so is not subject to thermal degradation. It has been assumed that the concentrations observed at harvest therefore persist throughout the supply chain until reaching the consumer. However, there are many factors that might affect phytochemical concentrations in rocket, such as preharvest factors, washing, drying, bagging, and duration and temperature of storage. These combined effects are likely to determine the health beneficial properties and the sensory quality of any bag of rocket purchased by the consumer.

#### 3.7.2 Shelf life modeling & volatile organic chemical markers for quality

A modeling study of *D. tenuifolia* shelf life conducted under simulated conditions, found that increasing storage temperature negatively influences appearance attributes and vitamin C abundance (Amodio et al., 2015). It would be interesting for future experiments to be conducted under true commercial conditions, and achieve a ‘real-world’ picture of leaf quality. It would also be valuable to expand upon the types of phytochemicals analysed to include GSLs/GHPs and flavonols, which (arguably) would have greater implications for human health.

Bell, Spadafora, Müller, Wagstaff, and Rogers (2016) utilised a novel extraction and analysis method to observe volatile organic chemical (VOC) production of leaves throughout a seven day cold storage treatment (4 °C). It was determined that the VOC profile at the point of harvest was significantly different from later time points, measured at five and seven days post harvest. The relative abundances of GHPs within the headspace declined over time, with corresponding increases in dimethyl disulfide, sulfur volatiles and some green-leaf VOCs (a pattern also observed by Spadafora et al. (2016). Dimethyl disulfide in particular is often a characteristic compound associated with bacterial metabolism within rocket bags (Nielsen et al., 2008) and could be used as a marker for quality within the supply chain.

Other studies by Luca et al., 2016, Luca et al., 2017 have reported detailed experimental data on the effects of MAP on VOCs released from rocket leaves. These studies have noted that an oxygen level below the lower oxygen limit (LOL; where anaerobic respiration dominates) the largest limiting factor of shelf life is aroma VOCs. Conversely, if oxygen levels are above the LOL it is appearance that is the limiting factor. These observations are in agreement with modeling performed by Amodio et al. (2015). It is therefore apparent that a fine balance must be struck when utilising MAP for rocket salad to avoid the generation of off odours and/or the yellowing of leaves (Nielsen et al., 2008).

#### 3.7.3 The role of bacterial & leaf metabolism: interactions with glucosinolate biosynthesis

Bacteria are attributed with producing unpleasant odours within salad bags (Løkke et al., 2013). *E. sativa* extracts have been reported to show little antimicrobial activity (Ombra et al., 2017), and so mitigation of off odours could be achieved through improved storage conditions within the supply chain to inhibit bacterial growth, rather than through increased GSL content as has been hypothesised in this context. An experiment by Bell, Yahya et al. (2017) within the supply chain also indicated that GSL and GHP abundance have little impact upon microbial growth. GHPs were seen to increase significantly over a nine-day postharvest time period. In that study it was found that immediately after field harvest, GSL concentrations were typically low, but subsequently increased post-processing and during shelf life, along with bacterial numbers. The average concentration of GSLs at harvest in the five *E. sativa* cultivars tested was 2.4 mg.kg^−1^ dw, but by day seven of cold storage shelf life (SL7) this amount had recovered to 7.1 mg.kg^−1^ dw (Table 2). A similar pattern was also observed for SF production, which increased significantly during shelf life storage.

The concentrations of free amino acids (particularly glutamine) also increased over the same period, inferring increased protein breakdown (Buchanan-Wollaston et al., 2003). The production of ammonia has also been highlighted as an important compound in this process (Mastrandrea et al., 2016), and is indicative of leaf senescence (Cantwell et al., 2010). Bell, Yahya et al. (2017) also noted a strong correlation between bacterial load of leaves and the abundance of free amino acids and GSLs. While this is not proof of a causal relationship, combined with what is known of GSL-metabolising bacteria within soil and the phyllosphere (Hanschen et al., 2015, Albaser et al., 2016), it may be that endemic bacteria on rocket leaves have a mechanism for metabolizing GSLs as a source of carbon and/or sulfur; possibly by utilising myrosinase-like enzymes.

Conversely, microbes may elicit a defensive response, causing an up-regulation of GSL biosynthesis genes. More studies are needed to verify which mechanisms are the driving factors. If bacterial strains could be isolated from rocket leaves using similar methods to Albaser et al. (2016), this would help to determine if there is a direct link with GSL biosynthesis, and if there is a significant myrosinase-like enzymatic defense system that would allow tolerance and/or metabolism of GSLs and GHPs.

#### 3.7.4 Genetic markers for post harvest quality

A study by Cavaiuolo, Cocetta, Bulgari, Spinardi, and Ferrante (2015) identified potential molecular markers for monitoring the postharvest quality of *D. tenuifolia* leaves. It was found that the expression of stress response genes *DtNAC3* and *DtStAR* were associated with reduced quality over the course of simulated shelf life. In a follow-up study by Cavaiuolo et al. (2017) an important resource of transcriptome sequences of plants under various stresses was established. The authors exposed plants to pre and post harvest stresses, which included salinity, heat, nitrogen starvation, cold, dehydration, darkness and wounding. All of these aspects are commercially relevant and the study identified hundreds of genes that are differentially expressed under the varying conditions.

Such studies could prove to be useful in screening crops during shelf life for specific genes, however they are limited from a crop improvement perspective as many of the proposed screening methods for quality are retrospective. The information is not explicitly being used to inform breeding selections and improve cultivars, and so integration into a crop improvement programme would be cumbersome. As only one rocket cultivar was tested in each respective study, it is also difficult to determine how representative of the species these data are. There are hundreds of rocket cultivars and landraces that are grown for the purpose of supplying commercial retailers. Indeed, some may perform markedly ‘better’ or ‘worse’ if tested under the same conditions, and thereby have given very different results. A single cultivar cannot therefore be taken as representative of the whole species, but such approaches do provide a large amount of data with which to proceed and scrutinise the genetics of the species in much greater detail.

Future genomic and transcriptomic analyses should therefore utilise more than one cultivar – the more phenotypically different from each other, the better – so that a range of responses can be determined. Finally, these experiments should have a downstream goal of informing breeding programs, so that enhanced phytochemical, sensory, and quality traits can be stabilized and improved. This should involve the generation of elite breeding material and mapping populations to determine the best genomic profile for enhanced traits. It is unlikely that any one gene is responsible for the complex taste, flavour, health benefits and processability of rocket, and so the recombination and expression of numerous genes is fundamental for developing informative breeding markers.

### 3.8 Reported in vitro & in vivo effects of rocket-derived phytochemicals

#### 3.8.1 General

Rocket species are often attributed with health beneficial effects, yet this is mostly inferred from what is known about related crops. Papers reporting ‘antioxidant effects’ of rocket extracts on cell cultures will not be highlighted or discussed here, as these such studies provide no meaningful mechanism of action for the prevention or treatment of chronic illnesses.

#### 3.8.2 Flavonols

A mouse model by Fuentes, Alarcón, Fuentes, Carrasco, and Palomo (2014) has suggested that *E. sativa* extracts have beneficial cardiovascular effects. It was demonstrated that platelet aggregation and activation were decreased, and that inflammatory mediators were reduced, leading to fewer thrombus formations. The authors speculated that these effects may be due to concentrations of polyglycosylated flavonols present within rocket, however no data were presented for the concentrations of these compounds within extracts.

A paper by Kishore et al. (2018) found that kaempferol extracted from *E. sativa* seeds lessened behavioral and biochemical changes associated with diabetic neuropathy in rats, and reversed pain response. While the properties of rocket derived kaempferol are interesting, this is not a form in which the molecule is found *in planta*. Flavonols are present in high concentrations in the form of glycosides, not aglycones, and so these data are somewhat limited in their inferences to human consumption patterns and potential health-related uses. In order for such studies to be meaningful, a cross-disciplinary approach must be taken that accounts for the total phytochemical composition of leaves.

#### 3.8.3 Isothiocyanates

Azarenko, Jordan, and Wilson (2014) conducted a study focused on erucin and its application to human MCF7 breast adenocarcinoma cells. It was observed that cell proliferation was inhibited (IC_50_ = 28 μM) and cell cycle arrest at mitosis initiated (IC_50_ = 13 μM) by the compound. This effect was due to interference by erucin in the formation of microtubules during cell division; a mechanism that the authors state is qualitatively similar to SF, and some microtubule-targeting anti-cancer drugs, such as eribulin, maytansinoids, taxanes, and vincas. There is a 1000-fold-potency difference between these chemotherapy drugs and ITCs, but a key benefit of erucin and SF is that they are not toxic to humans when ingested regularly in the diet. There are however no long-term chronic studies that have specifically looked at the effects of consuming these compounds, and so the significance they have on human health over long periods (years and decades) is unknown.

It is well established that SF has the capacity to affect carcinogenesis at several stages through interaction with several cellular mechanisms (Tortorella et al., 2015). ITCs are metabolized via conjugation with glutathione, mediated by glutathione-S-transferase (GST), and impart beneficial health effects *in vivo*. Recent studies have shown that SF can prevent and suppress tumor formation by affecting epigenetic changes, reversing errors in gene transcription, demethylation, and the modulation of microRNAs (Tortorella et al., 2015).

Some of the most recent research into the health benefits of SF has shown that another mechanism of action may be through interference with long noncoding RNAs (lncRNAs). These were differentially expressed in prostate cancer cells when SF was applied (15 μM), and correlated with genes involved in cell cycle regulation, metabolism and signal transduction. The work by Beaver et al. (2017) linked this genetic interaction with the long noncoding RNA LINC01116, and determined that it has an oncogenic function, and may therefore be suggestive of a mechanism by which dietary SF could prevent prostate cancer formation.

This follows and compliments previous work conducted by Traka et al. (2008) that showed ITC interactions with different *GSTM1* genotypes and oncogenic signaling pathways in the prostate. The presence of significant amounts of SF in rocket, as evidenced by Bell et al., 2017, Ku et al., 2016, means that such mechanisms could also be applicable to its ingestion, as with broccoli. As plants do not require cooking, more SF derived from rocket could theoretically be available and effective at eliciting such effects *in vivo*, and would suffer little (if any) myrosinase breakdown or thermal degradation preceding consumption.

#### 3.8.4 Nitrate and nitrite

Rocket species are known to accumulate high concentrations of nitrate, and it has been speculated that this may be both a health concern and a health benefit to humans. The former hypothesis has received little experimental evidence to support it (such as associations with gastric cancer; Ashworth et al., 2015); however the latter hypothesis has some evidence, particularly relating to sports nutrition. It has been hypothesised that dietary nitrate found in crops such as rocket have beneficial cardiovascular effects, and may be of use as dietary supplements to athletes to reduce blood pressure and increase performance. A study by Jonvik et al. (2016) demonstrated that rocket leaves could effectively increase plasma nitrate and nitrite, and reduce blood pressure significantly. Such studies may only be of niche importance however, as the rocket was consumed as a concentrated beverage – a form that is not common, and unlikely to be palatable or acceptable to everyday consumers. Future work should focus on the consumption of whole leaves and at relevant dietary concentrations.

## 4 Future research

### 4.1 Responses to biotic & abiotic stress

#### 4.1.1 Climate & environment

As rocket species are relatively unstudied when compared to crops such as *B. oleracea*, there are numerous avenues for further study. Perhaps one of the key areas is in understanding how environmental stresses and stimuli impact upon phytochemical composition. Numerous studies have characterised chemical constituents within one environment, but few have contrasted with multiple ones to determine the degree of the effects. The specifics of how preharvest temperatures and conditions impact rocket sensory quality and GSL accumulation are undocumented within the literature, as no robust replicated experiments have been conducted between countries/regions/climates.

Rocket species are Mediterranean crops, but are grown on every inhabited continent. As such, conditions vary enormously. It remains unknown how cultivars respond to different climatic conditions, or indeed to changes in season. The effects of light intensity have been explored in just two varieties of commercial rocket (Jin et al., 2009), but the effects of different wavelengths have not been examined. These factors may have broad implications for commercial productions, potential health benefits, and sensory attributes of rocket crops.

#### 4.1.2 Nutrient application

The application of supplementary sulfur to rocket plants is another area that has not been well explored in the literature to-date. Sulfur is essential for plant defense compounds (Capaldi et al., 2015), and modifying the supply in commercial crops could potentially enhance the health-related properties of rocket. In other Brassicaceae species it is well known that sulfur application influences GSL accumulation, often elevating the compounds significantly (Schonhof et al., 2007).

Increased nitrogen application by comparison has been shown to lower GSL concentrations in rocket (Chun et al., 2015). With spurious seasonal limitations on the amount of nitrate present within rocket leaves imposed by European regulation 1258/2011 (7000 mg.kg^−1^ fw, October to March; 6000 mg.kg^−1^ fw, April to September; Cavaiuolo & Ferrante, 2014), it would seem prudent for growers to limit the amount of nitrogen supplied to rocket crops. This would ensure that regulations are not breached, but also have the added benefit of potentially enhancing GSL compounds within leaves. More rigorous scientific studies are required with an increased number of cultivars in order to determine the specific effects supplementation has on rocket GSL profiles. The determination of any adverse health effects (if any) associated with high nitrate has also yet to be determined, and requires significant clinical experimental validation.

The effects of other nutrients and elicitors such as selenium and methyl jasmonate are as yet unstudied in rocket species. Their application in other Brassicaceae has yielded mixed results in terms of promoting GSL biosynthesis and health-related properties (Kim & Juvik, 2011), but these may still be areas for exploration in rocket crops.

#### 4.1.3 Multiple harvests

Despite the fact that commercial rocket crops are routinely harvested multiple times, little research has been conducted in order to determine how GSL and GHP profiles change as a result of this process. It is known anecdotally that leaves increase in pungency, but whether this is due to an increase in total GHP abundance, or an increase in only specific compounds, has not been demonstrated.

The known impacts upon the plant phytochemistry, as a result of multiple harvests, also include increases in production of green-leaf volatiles such as (*E*)-2-hexenal. These act as part of defense mechanisms of the plant and associated genes are known to be heavily up-regulated (D’Auria et al., 2007). These have effects upon both insect pests and microorganisms, but in terms of postharvest quality may influence crop taste and flavour.

Insect herbivore attacks are known to induce GSL accumulation, and the harvest process may also initiate this in rocket (Mewis et al., 2012). The activation of WRKY transcription factors and the action of jasmonates and abscisic acid are well known to regulate plant defense responses (Phukan et al., 2016), and research into the genetics of rocket species would enable greater understanding of secondary metabolite accumulations. Investigation of such harvest effects would be simple in experimental terms, but as with all aspects, would require the testing of multiple cultivars across multiple environments.

### 4.2 Relationships with endemic soil & leaf bacteria

Endemic soil and leaf bacteria may play a role in influencing rocket GSL profiles. Recently, species of GSL-metabolizing bacteria (*Citrobacter* spp.) have been isolated and cultured from soil, and some of which use sinigrin as a carbon source (Albaser et al., 2016). Research into the effects of GSLs and GHPs within the soil medium have found that it is the intact GSLs impacting microbial growth, not the hydrolysis products (Hanschen et al., 2015), suggesting the presence of species using GSLs as a carbon source within the rhizosphere.

Studies in *Arabidopsis thaliana* have shown a link between soil bacteria and the assimilation of sulfur, which is in turn utilised by plants to produce GSLs (Aziz et al., 2016). Rocket species are similar in many physiological respects to *A. thaliana*, and translation of this research from the model organism to a commercial crop may reveal similar mechanisms, and perhaps a means with which to enhance/modulate GSL composition.

Other experiments have observed that bacteria endemic to the leaf surface may interact with plants to initiate GSL biosynthesis (Schreiner, Krumbein, & Ruppel, 2009). Conversely plants might respond to high levels of bacterial load through stress responses (Bell, Yahya et al., 2017). In either case, this is an intriguing area for future study that could elucidate hitherto unexplored plant-bacterial relationships.

### 4.3 Genome & transcriptome analysis

Cavaiuolo et al. (2017) have produced the only comprehensive transcriptome study of *D. tenuifolia*, identifying candidate genes associated with preharvest stresses for future study. Other studies have relied upon identification of homologous genes from *Arabidopsis* and *Brassica*, and provided little novel insight into rocket species. No full genome sequence is publically available for *Diplotaxis* or *Eruca* species at the present time.

In order to advance the development of rocket into the genomics era, genome and transcriptome sequences will need to be obtained for multiple, diverse cultivars. This will allow for novel gene discovery, and to properly elucidate the GSL biosynthetic pathways unique to the species in totality. It is likely that these may be quite different to those in previously studied crops, as the exact mechanism of glucosativin/DMB and diglucothiobeinin formation is as yet unknown (Agerbirk & Olsen, 2012). Transcriptome data obtained from different growth stages and during processing will also elucidate how the supply chain modifies GSL/GHP profiles. This is likely to be an expensive and time consuming process, but will be vital for understanding and breeding nutritionally and sensorially improved cultivars of rocket.

## 5 Conclusions

Rocket species are excellent candidates for health-related and sensory improvement through selective breeding. The diversity of morphological, phytochemical and sensory traits evidenced by previous research is an excellent resource for breeders to utilise to that end. As with broccoli GSL and GHP formation could be enhanced to produce cultivars with demonstrably greater health effects than regular types (Traka et al., 2013). That being said, rocket breeding is much farther behind *B. oleracea* species, as it has only come to prominence within the last 25 years. Commercial and packet rocket seed are not typically the products of dedicated breeding programs; rather they are simply the result of bulk propagation of landraces, with little intensive selection placed upon them for advanced traits. The resultant cultivars lack morphological uniformity, as well as phytochemical. Some breeding companies have active breeding programs and have produced some morphologically improved cultivars, but this has not yet extended to health beneficial or sensory components with any environmental stability. In order to achieve this, much more research into the genomics and transcriptomics of the species is required, and not just simply cherry-picking analogous genes from other, better-studied crops. Many genes may indeed be homologous, but the GSL profile of rocket is also very different from *Arabidopsis* and *Brassica*, with several unique compounds present. Novel gene discovery will therefore be needed in order to fully understand rocket GSL biosynthesis. The effects of the growth environment, nutrient application, and endemic soil and leaf bacteria may also need to be considered in greater detail in future studies.

Breeding selections should be conducted based on information from within the commercial supply chain, wherever possible. It is clear that harvest time, growth environment, processing and storage have significant impacts upon GSL and GHP concentrations. The end consumer should be the primary focus of selections for phytochemical traits and health benefits, as it is ultimately they who will consume the final product. This may seem an obvious statement, but researchers have not made this an overt consideration to-date.

An experimental aspect that is prevalent within the literature is the assumption that one rocket cultivar is reflective of whole species. This is clearly not one that can withstand scrutiny, as evidenced by studies of more than one accession (Bell et al., 2015, Ku et al., 2016). If shelf life VOC markers are to be identified, for example, several varieties must be tested, as the variation of volatile production has also been demonstrated to be high between genotypes (Bell et al., 2016). This is applicable to all aspects of rocket species evaluation, as there is no representative cultivar of all traits and environmental responses. This is well established for major commercial crops and needs to be adopted to avoid generalization about these highly diverse species.

The scope for improvement of rocket is huge, as consumer demand and increased interest in health beneficial foods is consistently rising. Research could benefit from adapting the roadmap set out for broccoli GSL improvement, and potentially be even more efficacious in producing health benefits due to its consumption as a raw, uncooked product. Increasing the health-related properties of rocket can only be achieved through consideration of the supply chain and shelf life conditions, and selecting for specific compounds such as glucoraphanin, glucoerucin, and their respective ITCs. Selecting for total GSL content is likely to adversely affect sensory profiles, and so should not be adopted without careful consideration of the preferences of the end consumer. Finally, clinical trials are needed to test any beneficial health effects of rocket, and to quantitatively compare these with other Brassicaceae species. Nutritional interventions and studies utilising relevant quantities of leaves in a form that is compatible with a daily diet (i.e. as leaves, not as a beverage) are also needed to properly assess their health-promoting effects.

## Funding

Dr. Luke Bell is supported by a BBSRC LINK award (BB/N01894X/1).
